# Supplementary figures and images for: The dynamic upper limit of human lifespan
Source: F1000Res. 2017 Jun 9;6:569. Originally published 2017 Apr 26. [Version 2] doi: 10.12688/f1000research.11438.2 (PMC6039923; doi:10.12688/f1000research.11438.2)

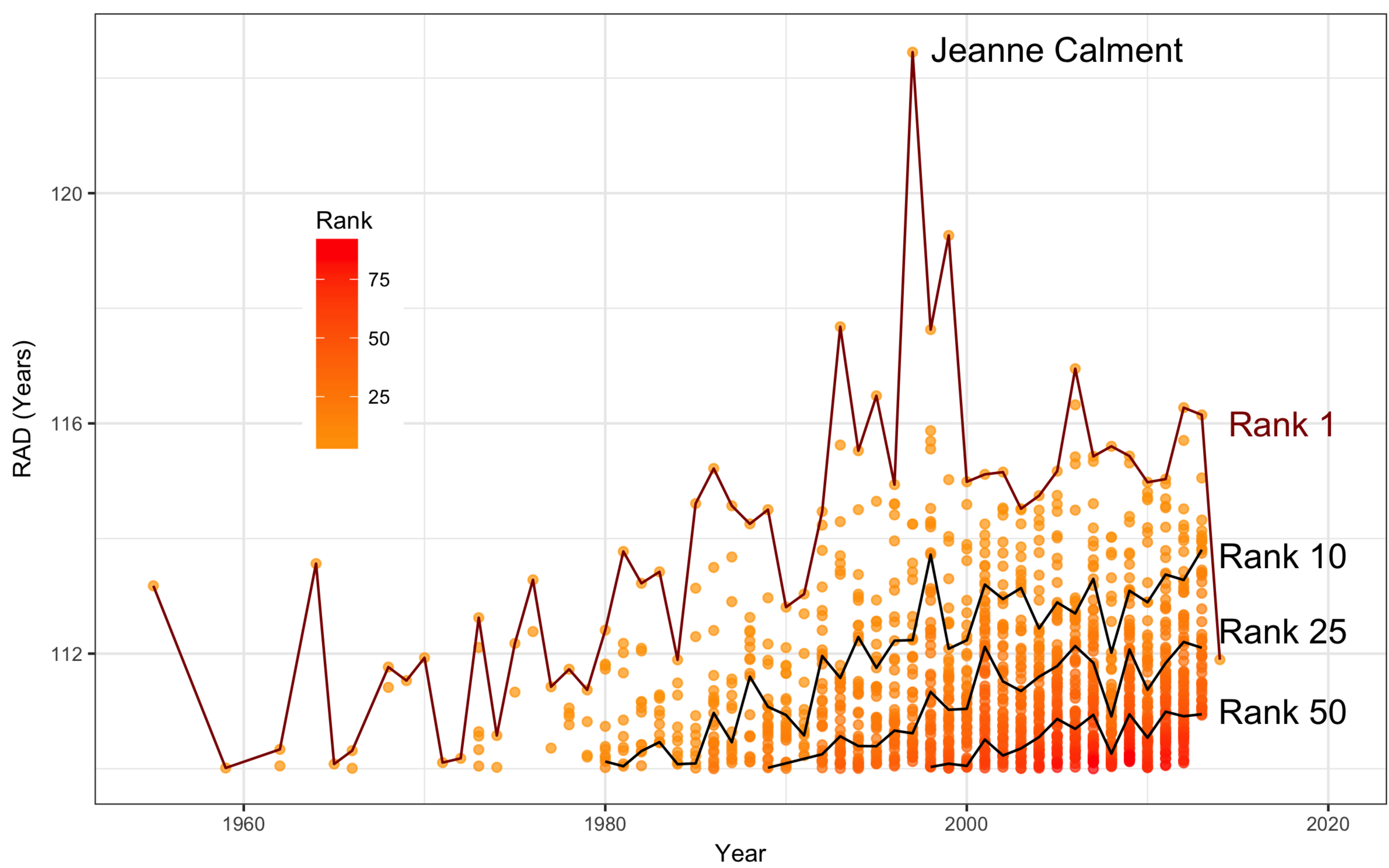

Supplement: Supplementary file 2 [file f1000research-6-12820-s0001.tgz › fc40e048-dce4-4946-9410-0331cd75d27a.pdf]

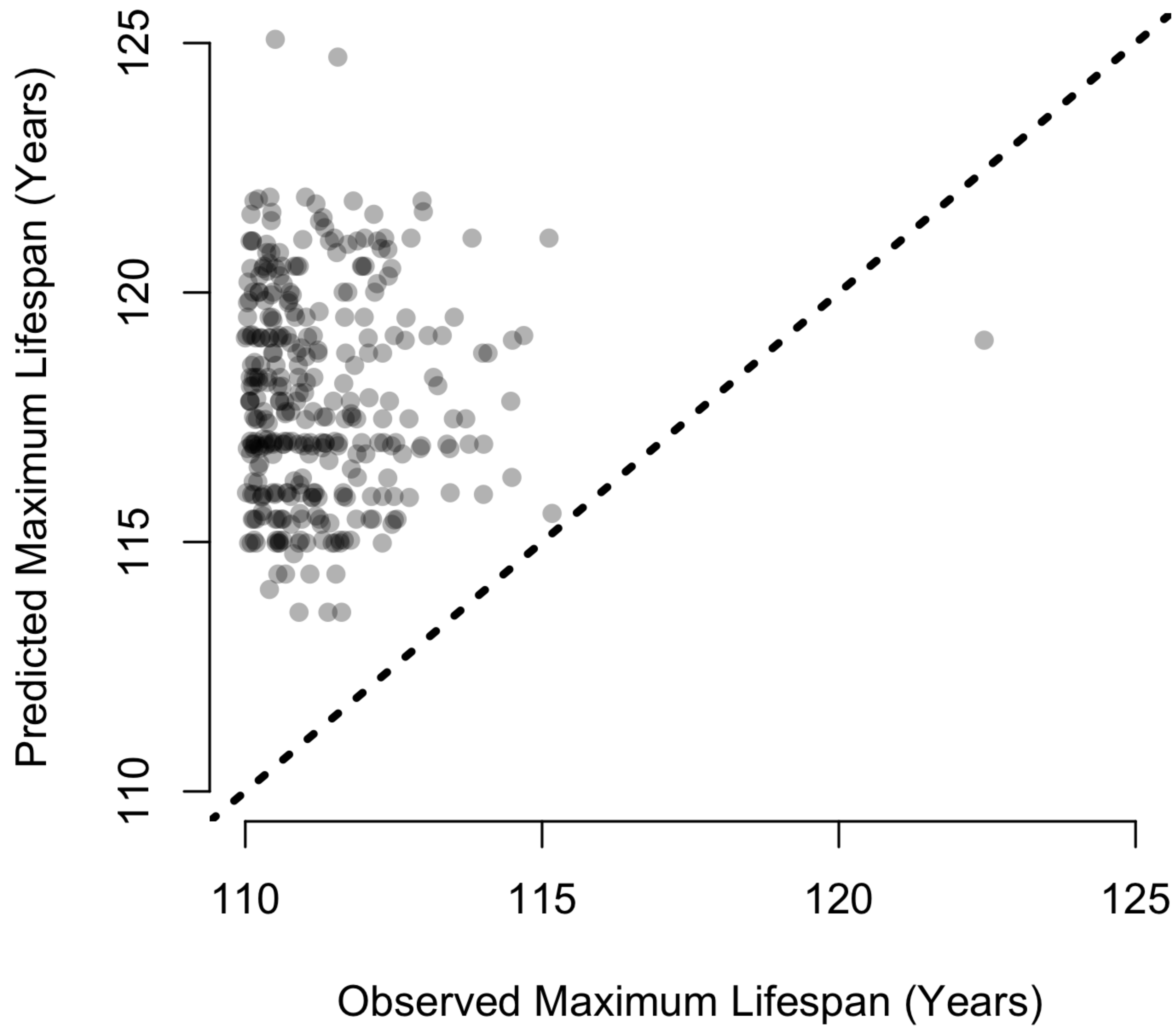

Supplement: Supplementary file 3 [file f1000research-6-12820-s0002.tgz › 7343b4d3-9ef7-490c-bacc-0f211e1f6723.pdf]
