# Supplementary material for: The dynamic upper limit of human lifespan
Source: F1000Res. 2017 Jun 9;6:569. Originally published 2017 Apr 26. [Version 2] doi: 10.12688/f1000research.11438.2 (PMC6039923; doi:10.12688/f1000research.11438.2)
Supplement: Peer review history

# Peer review: Referee response for version 2

**DOI:** 10.5256/f1000research.12820.r30810

**Published:** 2018-07-10

**Authors:** James Vaupel, José Manuel Aburto

**Affiliations:** 1 University of Southern Denmark, Odense, Denmark

Newman and Easteal ^1^ challenge the findings of Dong et al ^2^on the evidence of a limit for human lifespan. They highlight rounding errors and inappropriate selection of data. These errors have been documented in a series of communications to Nature ^3^^-^^6^ and in a recent article ^7^ questioning Dong et al’s results. Newman and Easteal provide code to reproduce their results, which is highly valuable.

 Newman and Easteal state that “we fit log-linear models to age-specific mortality rates”, which, called the Gompertz model, is common practice in demography for some ages (usually between 30 and 90). However, the authors do not fit a Gompertz model to the age-specific mortality rates (denoted m _x_ by demographers). Instead they fit log-linear models to the age-specific probabilities of death (which demographers denote by q _x_). They do so to estimate the Maximum Survivable Age (MSA), as can be seen from lines 950-989 of the provided code.

 There is a fundamental problem with this approach. A linear model fit to the log probabilities predicts probabilities exceeding one at high ages. To avoid this fallacy, Newman and Easteal postulate that the MSA is the age when their model predicts a probability of death of one. This is arbitrary and unconvincing, especially in light of evidence suggesting that age-specific probabilities of death reach a plateau at advanced ages at a value of about 0.5 ^8^^-^^9^. Although the authors acknowledge this evidence, they disregard it.

 Moreover, they fit their model using the Ordinary Least Squares approach, which assumes normally distributed errors that are identical over age. This is an assumption that does not hold, implying that their estimated coefficients and standard errors are not correctly calculated.

We have read this submission. We believe that we have an appropriate level of expertise to confirm that it is of an acceptable scientific standard, however we have significant reservations, as outlined above.

## References

[1] NewmanSEastealS: The dynamic upper limit of human lifespan. *F1000Research*.2017;6: 10.12688/f1000research.11438.2 10.12688/f1000research.11438.2 30026910PMC6039923

[2] DongXMilhollandBVijgJ: Evidence for a limit to human lifespan. *Nature*.2016;538(7624) : 10.1038/nature19793 257-259 10.1038/nature19793 27706136PMC11673931

[3] LenartAVaupelJ: Questionable evidence for a limit to human lifespan. *Nature*.2017;546(7660) : 10.1038/nature22790 E13-E14 10.1038/nature22790 28658239

[4] RozingMKirkwoodTWestendorpR: Is there evidence for a limit to human lifespan?. *Nature*.2017;546(7660) : 10.1038/nature22788 E11-E12 10.1038/nature22788 28658235

[5] de BeerJBardoutsosAJanssenF: Maximum human lifespan may increase to 125 years. *Nature*.2017;546(7660) : 10.1038/nature22792 E16-E17 10.1038/nature22792 28658213

[6] HughesBHekimiS: Many possible maximum lifespan trajectories. *Nature*.2017;546(7660) : 10.1038/nature22786 E8-E9 10.1038/nature22786 28658230

[7] RootzénHZholudD: Human life is unlimited – but short. *Extremes*.2017;20(4) : 10.1007/s10687-017-0305-5 713-728 10.1007/s10687-017-0305-5

[8] BarbiELagonaFMarsiliMVaupelJWWachterKW: The plateau of human mortality: Demography of longevity pioneers. *Science*.2018;360(6396) : 10.1126/science.aat3119 1459-1461 10.1126/science.aat3119 29954979PMC6457902

[9] GampeJ: Human mortality beyond age 110. In Supercentenarians. *Springer, Berlin, Heidelberg*.2010;219-230

# Peer review: Referee response for version 1

**DOI:** 10.5256/f1000research.12350.r22232

**Published:** 2017-05-30

**Authors:** Michael R. Rose, Laurence D Mueller

**Affiliations:** 1 Department of Ecology and Evolutionary Biology, University of California, Irvine, Irvine, CA, USA; 2 Department of Ecology and Evolutionary Biology, University of California, Irvine, CA, USA

Evidently the Dong et al. *Nature* article ^1^ made some major, though elementary, mistakes: (a) an incorrect rounding procedure; and (b) choosing data from an historical period (1900-1990) that generally fit a limited lifespan expectation, while neglecting to consider other data. Newman and Easteal ^2^ correct the rounding error and look at human cohort data from a broader range of historical dates. However, they fit log-linear Gompertz models to the data, though doing so is questionable as they themselves admit, if in fact human mortality rates plateau ^3^. It is often the case that extrapolating regression models beyond the range used to fit them is fraught with danger.

 Of most interest for us was whether the fundamental concept of inferring a species-wide maximum lifespan from one or even many cohorts of demographic data is cogent at all. With that in mind, we used mortality data from a twenty-cohort study of *Drosophila melanogaster *that we and our colleagues recently published ^4^. Specifically, we used “A-type” populations to predict sex-specific “maximum lifespans” for populations of “C-type” populations. Each of the ten pairs of A and C type populations share a common ancestral population in our laboratory, though they have since evolutionarily diverged.

 We took the initial sample size of, say, cohort CO-i, sex-j ( *N _ij_*), and then computed the age at which the probability of survival in the matching ACO-i, sex-j cohort is <= 1/(10* *N _ij_*).  We plotted the maximum lifespans predicted from the 20 “A” sex-specific cohorts versus the observed maximum lifespan in the 20 corresponding “C” cohorts (see Figure 1) using a double plateau Gompertz model ^4^. If the concept of species-wide maximum longevity were cogent, we would expect all the observed maximum lifespans to be near or well below the y=x line. In fact, most are well above that line, and show no correlation with the predicted values. Effectively, the maximum lifespan estimation procedure is not generally reliable. In the case of the example that we give here, the maximum lifespan was altered by substantial genetic changes caused by natural selection.

 We have long used experimental evolution to reconfigure the onset and end of periods of aging in carefully handled cohorts, as illustrated in publications 3 and 4. We do not regard maximum lifespans as characteristic of entire species, however they might be defined demographically. Rather, we view them as phenotypes that depend on both genotype and environment even in so-called “wild-type” populations, like most components of life history, as the extensive evolutionary literature on life history and aging has long suggested. More importantly, in cohorts that show a cessation of aging ^3^ we doubt that the concept of maximum lifespan has any biological cogency.

   

 Figure 1. The predicted maximum lifespan based on 10 A-type populations ^4^ and the observed maximum lifespan in 10 C-type populations:

https://f1000researchdata.s3.amazonaws.com/supplementary/11438/8c2e3298-9ed0-4ac5-8b85-ce62336a9bbb.png

We have read this submission. We believe that we have an appropriate level of expertise to confirm that it is of an acceptable scientific standard.

## References

[1] DongXMilhollandBVijgJ: Evidence for a limit to human lifespan. *Nature*.2016;538(7624) : 10.1038/nature19793 257-259 10.1038/nature19793 27706136PMC11673931

[2] NewmanSEastealS: The dynamic upper limit of human lifespan. *F1000Research*.2017;6: 10.12688/f1000research.11438.1 10.12688/f1000research.11438.1 30026910PMC6039923

[3] MuellerLDRauserCLRoseMR: Does Aging Stop?. *Oxford University Press*.2011;

[4] BurkeMKBarterTTCabralLGKezosJNPhillipsMARutledgeGAPhungKHChenRHNguyenHDMuellerLDRoseMR: Rapid divergence and convergence of life-history in experimentally evolved Drosophila melanogaster. *Evolution*.2016;70(9) : 10.1111/evo.13006 2085-98 10.1111/evo.13006 27431916

# Response

**Published:** 2017-06-08

**Authors:** Saul Newman

**Affiliations:** CSIRO Agriculture, Australia

We wish to thank MRR and LDM for their review. We agree with the ideas expressed, particularly with the risk of extrapolating log-linear models and the potential for improving estimates of lifespan limits using alternate models of old age survival.

There is little about maximum human lifespan or lifespan limits can be concluded with certainty. Therefore, our focus was on implementing the simplest available methods. The appended figure S2 supported the rationale for our use of log-linear models for projecting lifespan limits.

This figure reproduces the plot presented by MRR and LDM for *Drosophila* using human data. It plots the upper observed lifespan (MRAD) of individuals in the GRG against their calculated lifespan limit.

We could match 331 validated maximum lifespan observations to a historical gender-pooled MSA estimate. Of these, 330 approached but do not exceed their predicted limit. The only exception was Jeanne Calment, who outlived the gender-pooled MSA limit by 3 years but fell short of the female-cohort limit shown in Figure 3a. Unlike *Drosophila*, these calculated limits seem to fit reasonably well with MRAD.

However, there are many problems involved in human data that do not affect *Drosophila*. Human populations are not directly observable past their maximum age, individual variation in mortality acceleration is unknown, and ascertainment and validation problems abound.

Therefore, we still consider these projection methods, the GRG and IDL data, and much of the discussion of MRAD, problematic.

Finally, we agree that in species with a cessation of ageing or negative senescence there will be an unbounded, finite maximum lifespan with intrinsic limit. By extension, if there were a complete cessation of ageing in humans at extreme old ages there would be no intrinsic limit, but many probabilistic constraints, to human lifespan.

# Peer review: Referee response for version 1

**DOI:** 10.5256/f1000research.12350.r22231

**Published:** 2017-05-18

**Authors:** Jean-Michel Gaillard

**Affiliations:** 1 Laboratory of Biometrics and Evolutionary Biology(LBBE), CNRS (French National Center for Scientific Research), UMR5558, University of Lyon, Villeurbanne, France

This manuscript challenges the recent findings by Dong *et al*. that human lifespan has an upper limit and proposes that human lifespan is rapidly increasing. While I am quite convinced by all the problems the authors identified in the analysis performed by Dong *et al*. and by the maximum survivable age of about 125 years the authors estimated for humans, I am not convinced at all by the claim that maximum reported age at death is expected to rise over the next century.

 In particular, I do not understand the rationale of removing Jeanne Calment from the analysis. This data point has been validated. Contrary to the authors' interpretation, the mere existence of a lifespan of 122 observed 20 years ago without being even approached since then seems to indicate that some saturation in the maximal age at death is occurring. It is required to estimate the probability of not observing older ages at death for 20 years under different scenarios of increasing trends in maximal age at death.

 Detailed comments:

 p. 3 second column 3rd paragraph l. 1: Remove "significant"

 p. 3 second column 3rd paragraph l. 1: Should be "Calment", not "Clament"!

I have read this submission. I believe that I have an appropriate level of expertise to confirm that it is of an acceptable scientific standard, however I have significant reservations, as outlined above.

# Response

**Published:** 2017-06-06

**Authors:** Saul Newman

**Affiliations:** CSIRO Agriculture, Australia

We thank J-MG for these comments, and welcome the opportunity to clarify our analysis and expand our rationale for this study.

We wish to clarify that our projected increase in MRAD over the coming century is not based on IDL or GRG lifespan data, and these projections do not require either removal or inclusion of Jeanne Calment. Rather, these projections are based on theoretical lifespan limits calculated using HMD and WHO life table data, including the WHO population projections through 2100.

Our statement that “period data indicate that MSA is steadily increasing from a historic low c.1956 (Figure 3b and c) and that the MRAD is expected to rise over the next century” is based on observed HMD and WHO data for the “historic low” in 1956 and on WHO population projections over 2015-2100 for the ‘steady rise’. We can see that we did not make this distinction sufficiently clear and we have amended the text to make it clearer.

The rationale for removing Jeanne Calment from the regression of observed trends was to demonstrate her singular effect on recent MRAD trends. Jeanne Calment is a remarkable statistical outlier from historic trends in both Dong *et al*. and our linear models, with a Cook’s distance of 1.97 (Cook, 1982).

A large Cook’s distance is not necessarily grounds for ignoring a well-validated data point. Our finding that the inferred decline in MRAD is eliminated by removal of one outlier demonstrates that the inference is tenuous rather than invalid.

We find it notable that such broad conclusions on lifespan limits depend on the status of a single data point. We agree that the 20-year streak set by Jeanne Calment is remarkable, and we consider it valid. However, we disagree that this indicates a saturation of old-age survival.

Jeanne Calment’s record-holding streak is not unprecedented in length. Mary Kelly held the overall lifespan record for 17 years from 1964 to 1981. Gert Adrians-Boomgaard held the male lifespan record for 68 years. Mathew Beard broke this record, and held it for another 21.9 years (1985-2007). Like Jeanne Calment, Mathew Beard exceeded the next-lowest record by three years for this period.

We consider that these record-holding streaks do not reflect saturation of the MRAD, but the uncertain ascertainment and stochastic nature of lifespan records. Therefore, we suggest Jeanne Calment’s survival reflects a rare statistical event where survival has approached the calculated upper limit of lifespan in her cohort. We think this event is a result of stochastic variation, and is biasing the projection of short-term trends. The simulated removal of Jeanne Calment was intended to indicate this. 

More broadly, we feel that too much emphasis is placed on this single data point. Jeanne Calment’s predecessor Carrie White held a well-verified claim to the world’s oldest and then second-oldest woman for 24 years, before the claim was recognised as a clerical error in 2012 and retracted.

In the unlikely event that Jeanne Calment’s lifespan claim is also false, demographers would be depending on conclusions about lifespan limits from a single, false data point that is an outlier from the aggregate trend across 1626 other supercentenarians in the GRG (see figure S1).

Furthermore, a declining or flat trend in lifespan limits is inconsistent with our analysis of 3.3 billion lifespan records in 194 nations. We maintain that statistical trends in data for billions of deaths should not be ignored in favour of a single data point in 1997.

Finally, we accept our spelling error for Jeanne Calment’s name. Yet we do not understand the request to remove the word ‘significantly’ from the text: the negative trend was significant (p=0.03).
